# Editorial Comment to Nonmetastatic castration‐resistant prostate cancer treated with salvage focal brachytherapy after external beam radiotherapy

**DOI:** 10.1002/iju5.12344

**Published:** 2021-07-04

**Authors:** Yukiyoshi Hirayama, Taro Iguchi

**Affiliations:** ^1^ Department of Urology Osaka City University Graduate School of Medicine Kahoku Ishikawa Japan; ^2^ Department of Urology Kanazawa Medical University Kahoku Ishikawa Japan

Salvage local therapy for prostate cancer recurrence without metastatic disease includes radical prostatectomy (RP), brachytherapy, high‐intensity focused ultrasound, or cryosurgical ablation. Yoshida et al. reported local recurrence after external beam radiotherapy (EBRT), which was proven by prostate multiparametric MRI and re‐biopsy and treated with salvage focal brachytherapy.^1^ Compared to RP, high‐dose‐rate brachytherapy showed less severe genitourinary and gastrointestinal toxicity in a meta‐analysis of salvage therapy for recurrent prostate cancer after EBRT.^2^ A phase 2 study of salvage brachytherapy showed that the biochemical disease‐free survival, distant metastasis‐free survival, and cause‐specific survival rates were 68.5%, 81.5%, and 90.3% at 5 years, respectively.^3^ Focal brachytherapy is also an eligible option in highly selected patients. In this case, a relatively young age, long interval to recurrence, long PSA doubling time, and the recurrence detected as a solitary nodule in the ventral prostate are factors that might result in long‐term efficacy (23 months) and absence of side effects.

An earlier and more accurate characterization of disease is crucial to provide curable treatment options and improve outcomes in patients with biochemical recurrence. Advancing imaging techniques, such as Ga‐68‐PSMA positron emission tomography (PET), have the potential to change the current treatment strategy in the setting of biochemical relapse. As it has been reported that 33.3% of male patients had local recurrence after radiotherapy with PSMA‐PET below the Phoenix PSA threshold,^4^ we may need to re‐evaluate the current criteria for PSA recurrence after radiotherapy. Local recurrence, as well as metastasis‐directed therapy, will be increasingly important in the era of advanced molecular imaging. A larger prospective study is required to determine the selection of a local treatment modality.

## Conflict of interest

Taro Iguchi has obtained research funding from Bayer and Astellas, has served as a consultant or advisor from Bayer and Astellas, has participated in the speakers’ bureau for Bayer, Janssen, Sanofi and Astellas. Yukiyoshi Hirayama declares no conflict of interest.
